# Does COVID-19 Infection during Pregnancy Increase the Appearance of Congenital Gastrointestinal Malformations in Neonates?

**DOI:** 10.3390/biomedicines11123105

**Published:** 2023-11-21

**Authors:** Timea Elisabeta Brandibur, Nilima Rajpal Kundnani, Marioara Boia, Daciana Nistor, Daniel Milan Velimirovici, Leonard Mada, Aniko Maria Manea, Eugen Radu Boia, Marioara Nicula Neagu, Calin Marius Popoiu

**Affiliations:** 1 Department of Neonatology and Puericulture, “Victor Babes” University of Medicine and Pharmacy, 300041 Timisoara, Romania; 2 Department of Neonatology and Puericulture, “Louis Ţurcanu” Children Emergency Hospital, 300011 Timisoara, Romania; 3 Discipline of Internal Medicine and Ambulatory Care, Prevention and Cardiovascular Recovery, Department of Cardiology, “Victor Babes” University of Medicine and Pharmacy, 300041 Timisoara, Romania; knilima@umft.ro; 4 Discipline of Physiology, Department of Functional Sciences, Physiology, Center of Immuno-Physiology and Biotechnologies (CIFBIOTEH), “Victor Babes” University of Medicine and Pharmacy, 300041 Timisoara, Romania; daciana_nistor@umft.ro; 5 Centre for Gene and Cellular Therapies in Cancer, 300723 Timisoara, Romania; 6 Doctoral School, “Victor Babes” University of Medicine and Pharmacy, 300041 Timisoara, Romanialeo.mada@syonic.eu (L.M.); 7 Syonic SRL, 300254 Timisoara, Romania; 8 Department of Oto-Rhino-Laryngology, “Victor Babes” University of Medicine and Pharmacy, 300041 Timisoara, Romania; 9 Discipline of Physiology, Faculty of Bioengineering of Animal Resources, University of Life Sciences “King Mihai I”, 300645 Timisoara, Romania; 10 Department XI of Pediatric Surgery, “Victor Babes” University of Medicine and Pharmacy, 300041 Timisoara, Romania; mcpopoiu@yahoo.com

**Keywords:** COVID-19, neonates, gastrointestinal malformations, congenital anomalies

## Abstract

Background: COVID-19 was an infection that was capable of bringing the entire world to a standstill position within a period of days to months. Despite the advancements in the medical sector, the contagion was difficult to control and costed the lives of millions of people worldwide. Many short- and long-term effects are witnessed even to date in people that contracted the disease. Pregnant females had to suffer not only the devastating effects of the virus, but also the psycho-social impact of the lockdown. The impact of COVID-19 infection during pregnancy causing decreased antenatal care or hypoxemic episodes due to severe respiratory distress and whether it could lead to the appearance of congenital gastrointestinal malformation in neonates is still unclear. The aim of our study was to analyze if COVID-19 infection during pregnancy could increase the incidence of gastric malformations in neonates born from these women. Materials and Methods: We sifted the files of all neonates admitted into our hospital between January 2022 and December 2022, and based on inclusion and exclusion criteria, we included the cases having gastrointestinal congenital malformations during the COVID-19 pandemic. We performed a single-center, retrospective, observational descriptive study. We further divided the patients based on the anatomical location of the malformation. We also took down details of the evolution of pregnancy and whether the mother had contracted a SARS-CoV-2 infection during the pregnancy. Details regarding the Apgar score, days of intensive care admission, sex, and nutrition were the key findings studied. Results: A total of 47 neonates were found to have digestive anomalies, among which, based on the anatomical locations, the number of malformation cases found at the level of the esophagus were 15, while 16 occurred at the level of the pylorus; we found 12 cases of malformation of the duodenum, and four cases had malformation of the rectum. Out of these 47 neonates, 38.3% were females and 61.7% were males. A total of 58% were preemies, among which 9% had intra-uterine growth retardation (IUGR), and 42% were full-term newborns, among which 4% had intra-uterine growth retardation (IUGR). A total of 45% of the births were primiparous pregnancies and 55% were from multiparous females. A total of 14 mothers were found to have tested positive for COVID-19 during the course of pregnancy (*p*-value = 0.23); many had mild symptoms but were not tested. Conclusions: COVID-19 can affect the wellbeing of the pregnant female and their fetus. Larger studies can help gain extensive knowledge as to whether COVID-19 also has the potential to result in congenital gastrointestinal anomalies in children born from COVID-19 positive mothers. In our study, only a few infants born with this pathology were found to be born from COVID-19 positive mothers. Hence, it is difficult to conclude or exclude a direct correlation between the infection and the congenital malformations.

## 1. Introduction

COVID-19, caused by severe acute respiratory syndrome coronavirus 2 (SARS-CoV-2), spread quickly worldwide; this resulted in devastating effects on public health, affecting almost all organs and systems of human body, both during the acute phase of COVID-19 infection and during the immediate post COVID-19 period [1,2,3].

The risk of perinatal transmission, especially when breastfeeding, as well as the neonate’s risk of developing COVID-19 during the perinatal period are still unknown [4,5]. However, members of the coronavirus family are known to be responsible for severe complications during pregnancy, such as miscarriage, fetal growth restriction, and congenital anomalies [6]. Whether or not COVID-19 affects fetuses in the same way requires further in-depth studies. 

There is no reliable evidence for transplacental transmission of COVID-19 during the first or early second trimester of pregnancy; however, the current limited data does not indicate maternal-to-fetal transmission in the third trimester as well [7]. Meanwhile, a systematic review suggested that during delivery or while breastfeeding, the virus can enter the neonate and cause infection, but the chances of transplacental transmission are not yet documented [8]. During pregnancy, the maternal immune system and inflammatory responses are widely suppressed, and the fetus in the mother’s womb remains safe without the mother’s immune system attacking it, considering it a foreign entity [9]. Pregnant women were one of the most vulnerable groups during the COVID-19 pandemic, as pregnancy was found to be a strong risk factor for severity of COVID-19 infection [10]. 

Studies have shown that pregnant women may have an increased risk of maternal and neonatal complications due to COVID-19 infection [11]. Several clinical symptoms such as fever, disseminated intravascular coagulation, feeding intolerance, bleeding, cyanosis, complicated deliveries, rash, edema, dyspnea, and pneumonia have been reported in neonates born from mothers infected with COVID-19 [5,12,13]. Congenital anomalies include a wide range of anatomical or physiological abnormalities that can be present at birth or are diagnosed during the antenatal period. Primary prevention of congenital anomalies in the population, especially from the rural group, is of crucial priority, including pre-conceptional care and approaches involving the entire population in which education plays a pivotal role [14]. The urban population having easy access to healthcare units, awareness plans, and education helps immensely in maintaining the proper healthy state of pregnant women [15,16]. The global birth prevalence of congenital anomalies is approximately 2–3%. The pattern and prevalence of congenital anomalies may vary over time or with geographical location. Apart from the environmental factors, another key element is maternal age; the higher the age of the mother at the time of conceiving, the greater the chances of having an unhealthy child. Similarly, mothers suffering from chronic health issues often present stillbirths, low-birth weight infants, or miscarriages [17]. Proper antenatal care and deliveries at specialized units having neonatal intensive care units can further reduce the mortality. 

Early diagnosis of congenital malformations during the regular follow-up visits of the pregnant women can provide both the parents and doctors enough time to intervene and take proper decisions for the child and mother [18]. Ultrasound examinations are safe and non-invasive procedures that can diagnose many malformations in the fetus and the newborn [19,20]. The ability to diagnose these malformations prenatally is influenced by the site of obstruction, the presence of associated anomalies, and the gestational age at the time of imaging [21]. Newborns should be rapidly transferred to a tertiary medical care center that ensures adequate medical and surgical treatment if they were born in small medical units [22].

The published literature indicates that viral illnesses during early pregnancy and several antiviral drugs are associated with an increased risk of cardiac and neurodevelopmental congenital anomalies in newborns [23,24,25,26]. Similarly, over-the-counter medications like paracetamol together with other NSAIDs can have harmful effects during pregnancy [27]. However, there is very limited evidence for an association between SARS-CoV-2 infection in early pregnancy or COVID-19 vaccination and the risk of congenital malformations [24,28,29].

We have described 51 cases of newborns with GI pathology during a previous study [30]. The former study included 39 cases of GI malformations and spanned a period of 3 years (1 January 2017 up to 31 December 2019). During the year 2022, we noticed a spike in GI malformations, which prompted us to initiate the present study. 

The aim of the present study was to detect possible complications arising from COVID-19 infection. The focus was on gastrointestinal malformations and their relationship to a SARS-CoV-2 infection during pregnancy. The study was considered a research priority, motivated by the observed spike in GI congenital malformations. 

## 2. Materials and Methods

Newborns. This single-center retrospective descriptive analysis of birth prevalence for digestive malformations was performed during the year 2022 and included newborns admitted to the regional level III Neonatal Intensive Care Unit (NICU) of ‘Louis Turcanu’ Emergency Clinical Hospital for Children in Timisoara, Romania. The population-based data were collected from the “Atlas-Med” S.C. GAMA IT S.R.L, address is Str. Zidului nr. 7, Sibiu, 550189 (RO).

Inclusion criteria: gestational age (GA) ≥ 28 weeks, birth weight of at least 1000 g, surgery for malformations of the digestive tract, not more than 7 days old at admission, complete medical history from maternity and pediatric surgery department.

Exclusion criteria: GA < 28 weeks, birth weight under 1000 g, newborns without digestive malformations, incomplete observation sheets, severe infections (sepsis or pneumonia), severe genetic malformations, and postoperative deaths.

A full feeding was defined as the completion of target calorie counts for premature neonates (150 kcal/kg/day) [31]. We followed the national neonatal enteral and parenteral nutrition guidelines in our country. Although, there was a slow rate of nutritional recovery found in all newborns [32].

Ethical approval and patient consent: The study was approved by the Ethics Committee for Scientific Research of the Emergency Hospital for Children ‘Louis Turcanu’ (approval no. 82/05.10.2023). The authors ensure that this study was carried out in accordance with the Declaration of Helsinki. Written informed consent was obtained from all patients/parents/legal guardian as a part of routine admission to our tertiary university hospital for future research and study purposes.

Demographic variables and clinical data were collected (sex, GA, Apgar scores, antenatal clinic visit details, presence of COVID-19 disease during pregnancy, COVID-19 vaccination details during pregnancy, environment factors, birth weight, maternal medication, weight at admission and discharge, other associated diseases, number of hospitalizations in the pediatric surgery department and our department, postoperative nutrition, and details regarding the digestive malformation and its time of diagnosis).

Statistical analysis: Descriptive statistics are presented as frequencies or as the median and interquartile range (IQR). Groups were compared using the *t*-test or the Kruskal–Wallis rank sum test and using Fisher’s exact test or Pearson’s chi-square test. Pathologies were grouped into 4 categories based on the anatomical location: esophagus, pylorus, duodenum (including the small intestine), and rectum. The number of rectal malformations was small (4 cases), and this group was excluded from some of the analysis. Inter-group comparisons between the various pathologies were performed using analysis of variance (ANOVA), and the differences were highlighted using boxplots. Specific predictors were also analyzed in bivariate models to test if they remained statistically significant when confounded with the type of malformation. A *p*-value of <0.05 was considered to indicate a statistically significant difference. The statistical analysis was performed using the R statistical framework and plotted using the ggplot2 package [33,34,35].

## 3. Results

During the study period, 477 newborn babies were admitted into our department, out of which 57 had congenital malformations, corresponding to a prevalence of 12%. Out of the total number of malformations, seven were cardiac malformations (12.5%), three were renal system malformations (5.35%), and the remaining 82.25% of cases (*n* = 47) presented gastrointestinal (GI) malformations (upper and lower gut abnormalities). It is important to mention that our department is not part of a maternity hospital; we accept transfers from four counties in Romania with a wide variety of neonatal pathologies, and we work in close collaboration with the pediatric surgery department as being part of a tertiary pediatric multispecialty hospital.

The patients (*n* = 47) with GI malformations were further divided into four categories, based on the anatomical location: malformations at the level of the esophagus (*n* = 15), pylorus (*n* = 16), duodenum (*n* = 12), and rectum (*n* = 4). A total of 58% were preemies, among which 9% had intra-uterine growth retardation (IUGR), and 42% were full-term newborns, among which 4% had intra-uterine growth retardation (IUGR). 

The number of GI malformations was markedly higher during 2022 than during the 3 years prior to the COVID-19 epidemics (2017–2019, 39 cases, Fisher’s exact test *p*-value = 2.2 × 10^−6^). The total number of patients on the NICU was relatively stable during this time period (559, 626, and 678 cases vs. 679 cases during 2022). The increase in proportions was highly statistically significant (chi-square test trend in proportions: *p*-value = 1.2 × 10^−7^).

**Figure 1:**
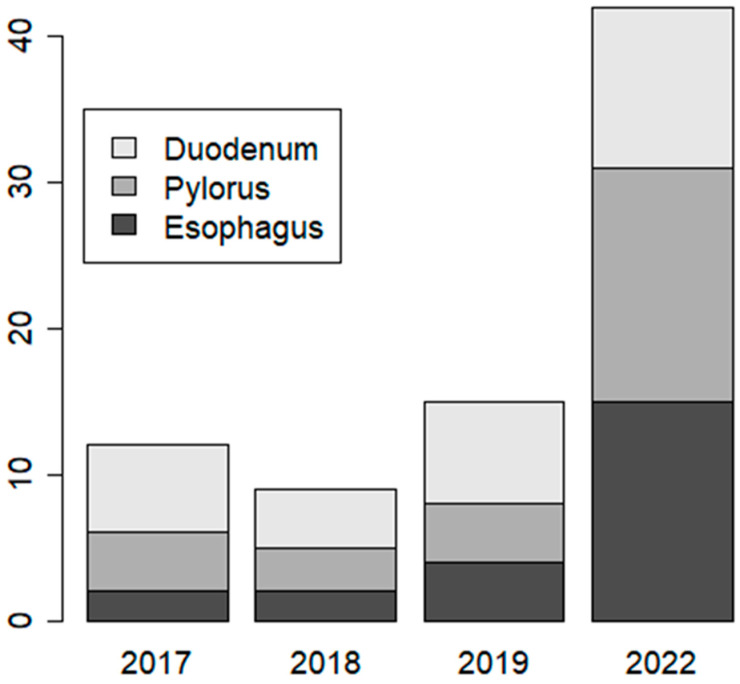
Bar plot with the number of GI malformations during the years 2017–2019 and 2022. There was a sharp increase during 2022. The number of patients was relatively stable during this time period. The increase in proportions was highly statistically significant. (*p*-value = 1.2 × 10^−7^).

The most common pathology during the previous 3 years corresponded to malformations of the duodenum (six, four, and seven cases; Figure 1), although the difference in the relative proportion did not reach statistical significance (chi-square test for trend in proportions: *p* = 0.06). Malformations of the rectum and colon occurred only infrequently during this time period (*n* = 3), a result which was also observed during 2022.

The second half of the study focused on the analysis of the 47 cases diagnosed during 2022. Almost half of the women were primiparous (45%), while the remaining 55% were multiparous.

The mothers were divided into three subgroups: those who had the COVID-19 disease (RT-PCR tested), those who did not have the disease, and the group of mothers who were not tested for IgG during pregnancy. Only a small number of mothers (approximately 13%) were found to be vaccinated against SARS-CoV-2. This could be due to limited knowledge regarding the disease and its potential side effects, as 51% of the mothers did not perform a COVID-19 test despite having mild symptoms indicative of a possible infection during the pregnancy. A history of COVID-19 disease could not be excluded in these women due to a lack of appropriate testing.

A positive history of COVID-19 (confirmed by RT-PCR) was present in 14 mothers, while another 8 did not experience an infection (negative RT-PCR). However, the status remained unknown in the remaining 25 mothers. Among the 14 COVID-19 positive females, only 1 had contracted the infection in the last trimester, while the remaining 13 had COVID-19 in their first trimester. 

During the study period, we assessed the antenatal medication and found out that all pregnant women who came from dispensary pregnancies supplemented their diet with nutrients and antioxidants to cover the increased needs during pregnancy; acetaminophen was the most used analgesic and antipyretic drug [27] (Table 1).

Out of the 47 analyzed cases, 61% were males (28 cases) and 39% were females. Abnormalities of the esophagus predominated in females (11 vs. 4), while those of the pylorus were more common in males (13 vs. 3). There were also seven males and four females with malformations of the duodenum, while all four cases with rectal malformations were males (Fisher test: *p* = 0.005). 

**Figure 2:**
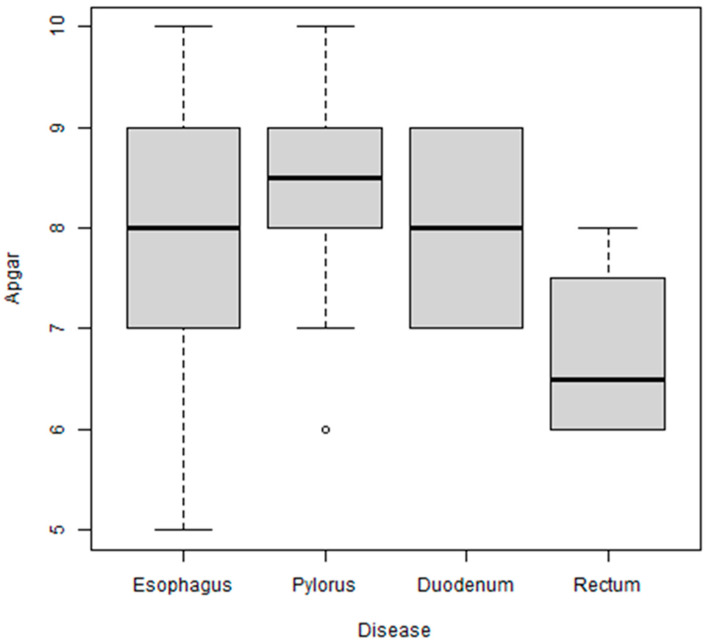
Boxplot with Apgar score grouped by pathology. The Apgar score was similar in the groups of patients with malformations of the esophagus, pylorus, or duodenum. There were only 4 patients with malformations of the rectum, with scores of 6, 6, 7, and 8. The difference did not reach statistical significance (*p* = 0.10), there was just one case having Apgar of 6 in the pylorus lot which is represented by a circle in the above figure.

The Apgar score at one minute was similar in the groups of patients with malformations of the esophagus, pylorus, or duodenum. It was slightly lower in the fourth group (rectal malformations), although the difference did not reach statistical significance (*p*-value = 0.10) (Figure 2). The mean scores (esophagus: 7.8; pylorus: 8.4; duodenum: 8.0; and rectum: 6.8) closely followed the medians (8, 8.5, 8, and 6.5 respectively). The Apgar score was lower in patients from a rural setting (mean = 7.52 vs. 8.39; *p*-value = 0.01).

**Figure 3:**
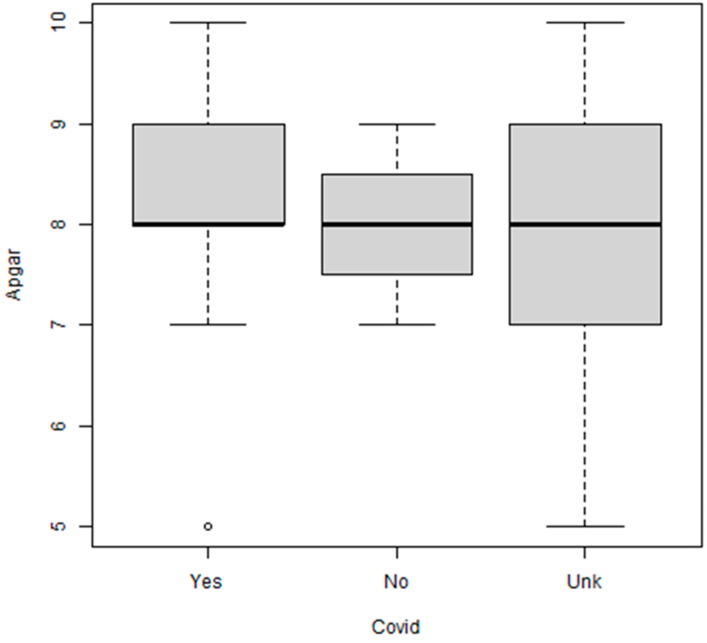
Apgar score and presence of COVID-19 infection. The Apgar score did not differ significantly with the COVID status of the mother (*p* = 0.70). However, there was a large number of mothers with unknown status (*n* = 24). Yes = infection; No = no infection; Unk = a previous infection could not be excluded. There was just one case having Apgar of 5 in the yes lot which is represented by a circle.

The Apgar score did not vary significantly with the COVID status (Kruskal–Wallis *p* = 0.70; Figure 3) even after merging the group with unknown status with the negative group (*p* = 0.41). The proportion of duodenal malformations was higher in pregnancies with a positive history for COVID-19 virus as well as in the untested group. However, these results did not reach statistical significance (*p* = 0.23; *p* = 0.76 in the merged groups).

Infection with COVID-19 during pregnancy had no impact on the following outcomes: gestational age (*p*-value = 0.57), weight at admission (*p*-value = 0.88), weight at discharge (*p*-value = 0.74), or Apgar score below 9 (*p*-value = 0.71).

Apgar score was found to be much lower in the patients with a rural background (*p*-value = 0.01). COVID-19 infection in mothers was found to have no influence on the gestational age (*p*-value = 0.57), weight at admission (*p*-value = 0.88), weight at discharge (*p*-value = 0.74), or Apgar score (below 9) (*p*-value = 0.71). 

The birth weight, weight at admission to the NICU, and discharge weight varied significantly with the underlying pathology (*p* = 0.01, *p* = 0.0005, and *p* = 0.005). Newborns with an esophageal malformation or duodenal malformation had lower birthweights compared with the other groups (medians of 2520 g and 2400 g compared with >3000 g for the pylorus and rectum). These results remained statistically significant in a multivariate analysis (Figure 3) and after removing the small group of rectal malformations. Sex did not reach statistical significance in the bivariate model for birthweight (*p* = 0.40) but did have an impact on the discharge weight, even when confounding for the underlying pathology (*p* = 0.033 for sex and *p* = 0.003 for pathology) (Table 2).

**Figure 4:**
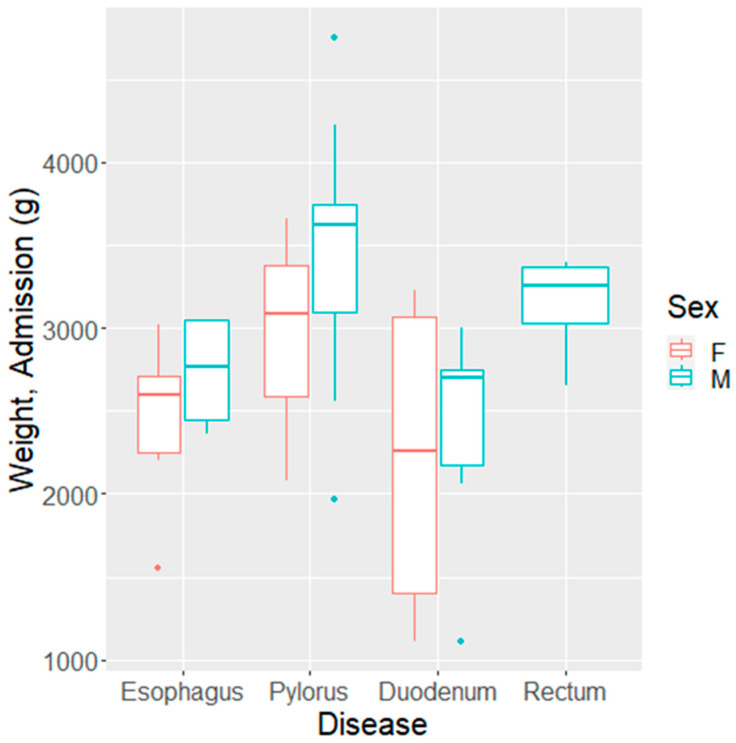
Boxplot of admission weight of newborns and their gastrointestinal malformations (F: females; M: males). The admission weight to the NICU varies with the type of gastrointestinal malformation and with sex. Females predominantly had abnormalities of the esophagus (11 vs. 4), while the pylorus was more commonly affected in males (13 vs. 3 cases). Males had also slightly more common malformations of the duodenum (7 vs. 4). All four infants with rectal malformations were males.

We observed that the proportion of duodenal malformations was higher in pregnancies with the COVID-19 virus and in the untested group. However, when evaluating the weight at admission and weight at discharge, it was found that all newborns born with digestive malformations were underweight (*p* = 0.01) (Figure 4).

**Figure 5:**
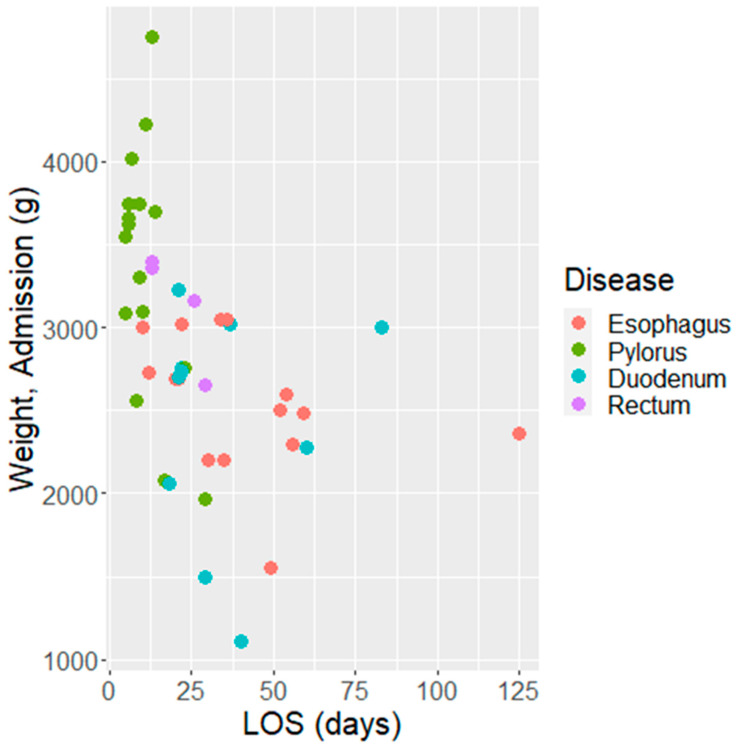
Total number of days of hospitalization depending on digestive malformations and weight at admission. The total number of days of hospitalization depends on the type of GI malformation and the weight at admission. Patients with malformations of the duodenum or esophagus had lower birth weights and were hospitalized longer (*p* < 0.001). The LOS in the NICU was lower for patients with malformations of the pylorus (*p* < 0.001, even in the bi-variate model) but did not differ with the COVID-19 status of the mothers (*p* = 0.30). The results did not change if the group with unknown COVID status was merged with the negative group and the group with malformations of the rectum was dropped (*p* = 0.43) (Figure 6).

The number of days of hospitalization varied both with the type of GI malformation and the weight at admission, as visualized in Figure 5. Patients with pyloric malformations had a much shorter duration of hospitalization compared with those with esophageal or duodenal malformations (median LOS of 9 days vs. 35 and 29 days, *p* = 0.0007). They also required fewer days in the NICU (median 4.5 days vs. 13 and 10 days). All patients, except those with malformations of the pylorus, required prolonged care in the neonatal ward following the discharge from the NICU as well (median 4 days for pylorus vs. medians of 14–19 days for the remaining types). The dataset also contains two outliers. Notably, one patient with a malformation of the esophagus required 125 days of hospitalization; the median LOS was stable at 34.5 days after excluding this patient.

Most of the patients were diagnosed antenatally (30 cases = 63.83%) during their dispensary/outpatient clinic visits, a fact that contributed to an early diagnosis and the approach of an effective therapeutic plan to favor the best possible evolution of the pregnancy. The children born from mothers who did not undergo follow-up during their entire pregnancy duration were diagnosed postnatally for their congenital digestive malformations (*n* = 15). 

## 4. Discussion

It is documented that SARS-associated coronavirus infections result in a high incidence of premature birth, miscarriages, or maternal deaths [36]. Other viral infections in early pregnancy (e.g., rubella) are well-recognized causes of specific anomaly syndromes as well [37]. The most robust evidence published previously is a population-based cohort study from Israel conducted by Goldshtein et al. that highlighted the same fact [21]. They found no evidence that singleton live births to women who were vaccinated in the first trimester had a higher risk of congenital malformations compared with those not exposed to vaccination in pregnancy [36]. We believe that even if the virus does not directly affect the normal growth and development of the embryo, other variables related to a COVID-19 infection can indirectly cause harm to the fetus. The factors that could possibly have a negative impact on the fetus health are decreased quantity, quality, and routine visits to the antenatal care units; poverty due to the COVID-19 pandemic and lockdowns; and shortage of fetal screening and diagnosis possibilities, especially during pandemic situations. Furthermore, there are studies that highlight the fact that congenital COVID-19 infection can lead to neurodevelopmental disabilities, mainly resulting in epilepsy, cerebral palsy, and neurosensory disorders [37].

Furthermore, to see the prevalence of gastrointestinal congenital malformations, we sifted the files of neonates admitted in our hospital during a three-year duration in the immediate pre-COVID-19 era. We found that a total of 39 patients were born with gastrointestinal malformations during the three-year study period, whereas from the current study, we had 47 cases registered with the same pathology in a mere one-year time duration, a result that was highly statistically significant (Fisher exact test: *p*-value < 0.001). This difference points out to a possible correlation between COVID-19 infection and congenital digestive malformations. The proportions were stable during the prior 3 years (chi-square trend in proportions *p*-value = 0.88), excluding an underlying systematic long-range trend. Due to a lack of data from other maternity homes in our city, the results obtained are quiet limiting. Furthermore, large cohort studies can provide clarity on this hypothesis. 

The mother’s stress levels hinder the development of the fetus [38]. The state of anxiety and the factors that influence it may differ depending on the severity of the outbreak in each geographic region and the access to the healthcare units during the lockdown [38,39]. Uncertainty regarding the best treatment and clinical management of patients with COVID-19 can affect both the mind and psyche of the pregnant women [39,40,41].

Furthermore, due to the modern sedentary lifestyle, more and more younger people are diagnosed with chronic diseases like obesity, dyslipidemias, arterial hypertension, diabetes mellitus, metabolic syndrome, and rheumatic disease and their cardio-vascular complications (stroke, myocardial infraction, chronic coronary syndrome, atrial fibrillation) as compared with the former times, where these were once considered to be diseases of advanced age [42,43,44] and were rarely present in women of childbearing age [23,24,25,26,45,46,47,48]. González V.S.E. et al. reported that obesity, diabetes mellitus, and arterial hypertension were associated with a higher risk of developing severe forms of COVID-19 infection (if contracted), accounting for the odds ratio regarding mortality (1.413 (IC 95%, 1.11–1.78)), obesity (1.753 (IC 95%, 1.39–2.20)), and diabetes mellitus and hypertension, respectively (1.961 (IC 95%, 1.57–2.45)) [49]. 

Thrombo-embolic complications, arrhythmic complications, and even acute heart failure may occur during or post COVID-19 infection and may impose the use of very complex therapeutic algorithms that may involve the use of oral or parenteral anticoagulants, anti-arrhythmic drugs (for the conversion of sinus rhythm for maintaining a proper heart rate), and renin–angiotensin–aldosterone system inhibitors [50,51]. Proper care should be taken while prescribing the treatment to pregnant females, keeping in mind not only the toxicity of the drugs but also that any sudden rhythm abnormality or hemodynamic changes can be fatal for the fetus. The anxiety and depression occurred in the acute phase or during the immediate post COVID-19 infection phase may necessitate the use of anxiolytics or antidepressive drugs, where again the safety of the fetus should be the prime priority [52]. Antibiotics were frequently used empirically and inadequately during the acute phase of COVID-19 infection, especially for severe cases with low oxygen saturation [53]. The use of such aggressive treatments in the periconceptual period or during the first weeks of pregnancy may result in multiple fetal malformations. This can frequently be seen in the case of unwanted or undiagnosed pregnancies in the initial phase of conception. 

Due to both the placental and perinatal hypoxic-ischemic events, the fetus is exposed to higher risks of developing congenital malformations or growth retardations [54]. Furthermore, the ischemic placental events are assumed to be correlated with intestinal atresia (more frequently localized on the jejunal, ileal, and colonic segments), but no statistical significance was found when the incidences were compared with the pre-COVID-19 era and COVID-19 pandemic era in the pregnant females having hypoxemic episodes due to COVID-19 infection [55]. The diagnosis of esophageal atresia is difficult to establish based on ultrasound, especially by obstetricians with no experience or competence in maternal-fetal medicine. Hence, the majority of cases are diagnosed after birth [56]. 

In this study, all digestive malformations operated during the pandemic period for one year were evaluated. No direct correlation could be concluded between digestive anomalies and COVID-19 infection during pregnancy. However, cases of duodenal malformations were higher in pregnancies from COVID-19 positive mothers. The mothers did not take antivirals, and there was no previous history of births with congenital malformations. 

Gastrointestinal malformations are often complicated by skeletal anomalies and intrauterine growth retardation [57]. We found statistically significant differences both in the case of admission weight and discharge weight in cases with digestive malformations, with the newborns being underweight. (*p*-value = 0.01) The average number of days of hospitalization in the intensive care unit was 16.2. Regarding premature babies or neonates suffering from certain conditions that occurred during the perinatal and neonatal period, newborns from the Neonatal Intensive Care Unit (NICU) have a high risk of developing neurological and developmental sequelae [58].

Furthermore, surgical infants can develop an aversion to oral feeding if oral feeding is delayed or if painful symptoms are associated with feeding, further hindering the growth and development of the child. Hence, clinical examination has a paramount role in the early detection of digestive malformations, in the effective management of specific necessary nutrition, and in the way of providing it [30]. Early trophic feeds may improve recovery time by increasing gut blood flow, improving motility and limiting the impact of starvation on the structure of the gut and its ability to absorb nutrients. Starting small-volume feeds of 10 mL/kg/day within 12–18 h of surgery may reduce the time needed until the full enteral nutrition is achieved [59]. Complicated cases (e.g., those with high stomas or extensive resection) may require either a hydrolyzed or lactose-free feed or a feed containing fats as medium-chain triglycerides (MCTs). The above-stated principles were followed for the best outcome in our hospital, keeping in mind the key elements of feeding in GI malformations [32,42]. 

The available data on vaccination against COVID-19 in pregnant women show that there is no specific cause for concern [32,59,60]. More data are clearly needed on the efficacy, safety, teratogenicity, and pharmacokinetics of drugs and biologics for pregnant and breastfeeding people with an active COVID-19 phase [61]. New agents are often licensed despite little information on key characteristics such as transplacental passage and drug labeling, which is unhelpful for informing clinical decisions for pregnant and breastfeeding people [62]. Clinical guidelines based on risk stratification for SARS-CoV-2 infection in children are needed to manage, monitor, and establish priority access for some groups to high medical care [63].

### Limitations in Our Study

Although we found out with certainty that 14 mothers were COVID-19 positive during pregnancy, there remains a large group of patients, a total of 24, who were not tested for IgG SARS-CoV-2, which does not exclude the possibility of the presence of the disease during pregnancy and possible correlations between COVID-19 and digestive malformations. Lack of confirmatory tests in these 24 cases is one of the limitations of the study. On the other hand, the small number of cases and the inability to compare the data of the study group with a control group is another limitation of the study.

## 5. Conclusions

The results of the study indicate that SARS-CoV-2 infection during pregnancy is unlikely to cause congenital digestive malformations; however, due to the small cohort studied, it would be inappropriate to generalize the findings and reach a conclusion. Nevertheless, no significant differences were witnessed with regard to the Apgar score, days of admission, or severity in children born to COVID-19 positive mothers compared with those born from COVID-19 negative mothers. However, we plan to conduct further studies on larger cohorts on RT-PCR-tested COVID-19 positive mothers, which can provide a better understanding. Let us not forget that pregnancy is monitored by obstetricians and gynecologists, while COVID-19 is managed by infectious disease specialists. Therefore, a multidisciplinary approach is a key to success when the timely management of information on the diagnosis and treatment of COVID-19 are necessary to avoid complications in newborns. Precise antenatal care can further improve the outcomes and help earn time for a timely management, especially during pandemics like the one just faced.

## Figures and Tables

**Figure 6:**
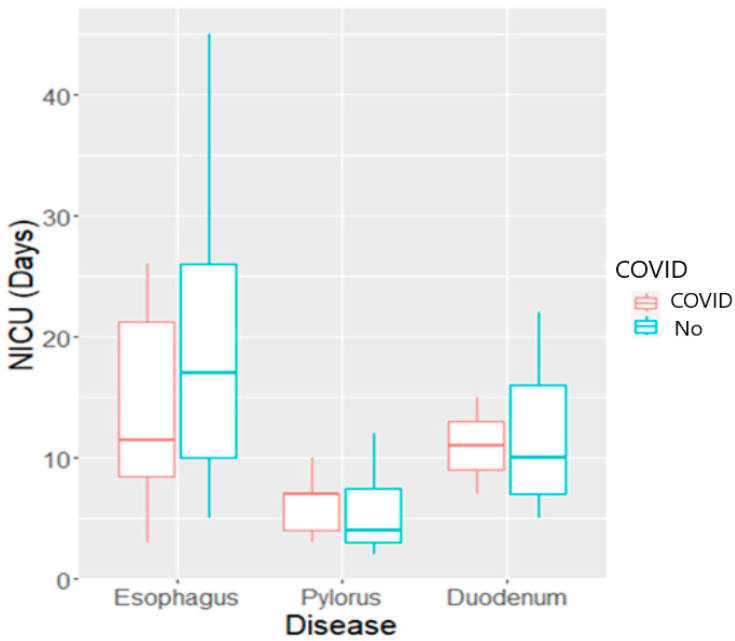
Boxplot of days of hospitalization in the intensive care unit grouped by COVID-19 and type of GI malformations. LOS in the NICU based on COVID-19 history of the mothers and type of GI malformation. The group with unknown COVID status was merged with the negative group.

**Table 1:** Demographic details of mothers and patients enrolled in the study.

	% or N (%)
Rural/Urban	50%/50%
Primipara (P/MP)	46%/54%
COVID-19 Status:	
Yes	14 (29.8%)
No	8 (17%)
Unknown	25 (53.2%)
COVID-19 Vaccine (Yes/No)	13%/87%
Vegan (Yes/No)	9%/91%
Sex (M/F)	61%/39%
Apgar Score at One Minute: <9 vs. ≥9	63%/37%
Nutrition (Diverse/Formula)	87%/13%

**Table 2:** Median (IQR) for the gestational age, weight at birth, admission and discharge, Apgar scores, LOS during ICU and post ICU, and total LOS of the patients included in the study.

Median (IQR)	
37 (36.0–38.0) weeks	Gestational Age
2750 (2190–3200) g	Birth Weight
2760 (2390–3282.5) g	Admission Weight
3135 (2600–3500) g	Discharge Weight
8 (7–9)	Apgar score
8 (5–13) days	LOS (ICU)
11.5 (5–21.75) days	LOS (post- ICU)
21.5 (11.25–35.75) days	LOS (Total)

## Data Availability

Data will be made available on justified request.

## References

[1] Zhu N., Zhang D., Wang W., Li X., Yang B., Song J., Zhao X., Huang B., Shi W., Lu R. (2020). Novel Coronavirus from Patients with Pneumonia in China, 2019. N. Engl. J. Med..

[2] Mocanu V., Bhagwani D., Sharma A., Borza C., Rosca C.I., Stelian M., Bhagwani S., Haidar L., Kshtriya L., Kundnani N.R. (2022). COVID-19 and the Human Eye: Conjunctivitis, a Lone COVID-19 Finding—A Case-Control Study. Med. Princ. Pract..

[3] Rosca C.I., Branea H.S., Sharma A., Nicoras V.A., Borza C., Lighezan D.F., Morariu S.I., Kundnani N.R. (2023). Rhythm Disturbances in Post-Acute COVID-19 Syndrome in Young Men without Pre-Existing Known Cardiovascular Disease—A Case Series. Biomedicines.

[4] Chen H., Guo J., Wang C., Luo F., Yu X., Zhang W., Li J., Zhao D., Xu D., Gong Q. (2020). Clinical characteristics and intrauterine vertical transmission potential of COVID-19 infection in nine pregnant women: A retrospective review of medical records. Lancet.

[5] Zhu H., Wang L., Fang C., Peng S., Zhang L., Chang G., Xia S., Zhou W. (2020). Clinical analysis of 10 neonates born to mothers with 2019-nCoV pneumonia. Transl. Pediatr..

[6] Di Mascio D., Khalil A., Saccone G., Rizzo G., Buca D., Liberati M., Vecchiet J., Nappi L., Scambia G., Berghella V. (2020). Outcome of coronavirus spectrum infections (SARS, MERS, COVID-19) during pregnancy: A systematic review and meta-analysis. Am. J. Obstet. Gynecol. MFM.

[7] Yang H., Wang C., Poon L.C. (2020). Novel coronavirus infection and pregnancy. Ultrasound Obstet. Gynecol..

[8] Sánchez-García J.C., Moreno N.P.C., Tovar-Gálvez M.I., Cortés-Martín J., Liñán-González A., Olmedo L.A., Rodríguez-Blanque R. (2022). COVID-19 in Pregnant Women, Maternal—Fetal Involvement, and Vertical Mother-to-Child Transmission: A Systematic Review. Biomedicines.

[9] Prabhudas M., Bonney E., Caron K., Dey S., Erlebacher A., Fazleabas A., Fisher S., Golos T., Matzuk M., McCune J.M. (2015). Immune mechanisms at the maternal-fetal interface: Perspectives and challenges. Nat. Immunol..

[10] Rasmussen S.A., Jamieson D.J. (2022). COVID-19 and Pregnancy. Infect. Dis. Clin. N. Am..

[11] Yang Z., Wang M., Zhu Z., Liu Y. (2020). Coronavirus disease 2019 (COVID-19) and pregnancy: A systematic review. J. Matern. Neonatal Med..

[12] Zamaniyan M., Ebadi A., Aghajanpoor S., Rahmani Z., Haghshenas M., Azizi S. (2020). Preterm delivery, maternal death, and vertical transmission in a pregnant woman with COVID-19 infection. Prenat. Diagn..

[13] Khan S., Jun L., Nawsherwan, Siddique R., Li Y., Han G., Xue M., Nabi G., Liu J. (2020). Association of COVID-19 with pregnancy outcomes in health-care workers and general women. Clin. Microbiol. Infect..

[14] Dolk H., Loane M., Garne E. (2010). The prevalence of congenital anomalies in Europe. Adv. Exp. Med. Biol..

[15] He R., Zhang J., Mao Y., Degomme O., Zhang W.-H. (2020). Preparedness and Responses Faced during the COVID-19 Pandemic in Belgium: An Observational Study and Using the National Open Data. Int. J. Environ. Res. Public Health.

[16] Bin Nisar Y., Aurangzeb B., Dibley M.J., Alam A. (2016). Qualitative exploration of facilitating factors and barriers to use of antenatal care services by pregnant women in urban and rural settings in Pakistan. BMC Pregnancy Childbirth.

[17] Patra C., Sarkar S., Dasgupta M., Nayek K., Karmakar P. (2013). Prevalence of congenital anomalies in neonates and associated risk factors in a tertiary care hospital in eastern India. J. Clin. Neonatol..

[18] Boyle M.I., Jespersgaard C., Brøndum-Nielsen K., Bisgaard A.M., Tümer Z. (2015). Cornelia de Lange syndrome. Clin. Genet..

[19] Liao Y., Wen H., Ouyang S., Yuan Y., Bi J., Guan Y., Fu Q., Yang X., Guo W., Huang Y. (2020). Routine first-trimester ultrasound screening using a standardized anatomical protocol. Am. J. Obstet. Gynecol..

[20] Menon P., Binu V., Rao K.L.N., Suri V. (2018). Trends in referral pattern of antenatally diagnosed surgical abnormalities in a tertiary care center in North India. J. Indian Assoc. Pediatr. Surg..

[21] Goldshtein I., Steinberg D.M., Kuint J., Chodick G., Segal Y., Ben David S.S., Ben-Tov A. (2022). Association of BNT162b2 COVID-19 Vaccination During Pregnancy With Neonatal and Early Infant Outcomes. JAMA Pediatr..

[22] Fung A.C.H., Kan A.S.Y., Chung P.H., Shek N.W.M., Chan I.H.Y., Wong K.K.Y. (2021). Antenatal counselling of congenital surgical anomalies: A decade of experience in a local tertiary centre. J. Paediatr. Child Health.

[23] Leung A.K., Hon K., Leong K. (2019). Rubella (German measles) revisited. Hong Kong Med. J..

[24] Hoffman M.C., Freedman R., Law A.J., Clark A.M., Hunter S.K. (2021). Maternal nutrients and effects of gestational COVID-19 infection on fetal brain development. Clin. Nutr. ESPEN.

[25] Faizan I., Abdullah M., Ali S., Naqvi I.H., Ahmed A., Parveen S. (2016). Zika Virus-Induced Microcephaly and Its Possible Molecular Mechanism. Intervirology.

[26] Luteijn J.M., Brown M.J., Dolk H. (2013). Influenza and congenital anomalies: A systematic review and meta-analysis. Hum. Reprod..

[27] Zafeiri A., Raja E.A., Mitchell R.T., Hay D.C., Bhattacharya S., A Fowler P. (2022). Maternal over-the-counter analgesics use during pregnancy and adverse perinatal outcomes: Cohort study of 151 141 singleton pregnancies. BMJ Open.

[28] Rasmussen S.A., Smulian J.C., Lednicky J.A., Wen T.S., Jamieson D.J. (2020). Coronavirus Disease 2019 (COVID-19) and pregnancy: What obstetricians need to know. Am. J. Obstet. Gynecol..

[29] Heidarzadeh M., Taheri M., Mazaheripour Z., Abbasi-Khameneh F. (2022). The incidence of congenital anomalies in newborns before and during the COVID-19 pandemic. Ital. J. Pediatr..

[30] Brandibur T.E., Manea A.M., Sharma A., Kundnani N.R., Popoiu M.C., Ahmad B., Dahdal D.S., Cioboata D., Lungu N., Doandes F.M. (2022). Macronutrients Management for Growth in Neonates with Congenital Gastrointestinal Malformation. Experiment.

[31] Ben X.-M. (2008). Nutritional management of newborn infants: Practical guidelines. World J. Gastroenterol..

[32] https://old.ms.ro/?pag=181.

[33] R Core Team (2021). R: A Language and Environment for Statistical Computing.

[34] Wickham H. (2009). ggplot2: Elegant Graphics for Data Analysis.

[35] Bland M. (2000). An Introduction to Medical Statistics.

[36] Yu W., Hu X., Cao B. (2021). Viral Infections During Pregnancy: The Big Challenge Threatening Maternal and Fetal Health. Matern. Med..

[37] Ravaldi C., Wilson A., Ricca V., Homer C., Vannacci A. (2020). Pregnant women voice their concerns and birth expectations during the COVID-19 pandemic in Italy. Women Birth.

[38] Ayaz R., Hocaoğlu M., Günay T., Yardımcı O.D., Turgut A., Karateke A. (2020). Anxiety and depression symptoms in the same pregnant women before and during the COVID-19 pandemic. JPME.

[39] Tomfohr-Madsen L.M., Racine N., Giesbrecht G.F., Lebel C., Madigan S. (2021). Depression and anxiety in pregnancy during COVID-19: A rapid review and meta-analysis. Psychiatry Res..

[40] Liu C.H., Erdei C., Mittal L. (2021). Risk factors for depression, anxiety, and PTSD symptoms in perinatal women during the COVID-19 Pandemic. Psychiatry Res..

[41] Andersson C., Vasan R.S. (2017). Epidemiology of cardiovascular disease in young individuals. Nat. Rev. Cardiol..

[42] Wang X., Liu J., Cheng Z., Zhong Y., Chen X., Song W. (2021). Triglyceride glucose-body mass index and the risk of diabetes: A general population-based cohort study. Lipids Health Dis..

[43] Conklin A.I., Guo S.X., Tam A.C., Richardson C.G. (2018). Gender, stressful life events and interactions with sleep: A systematic review of determinants of adiposity in young people. BMJ Open.

[44] Fortin O., Mulkey S.B. (2023). Neurodevelopmental outcomes in congenital and perinatal infections. Curr. Opin. Infect. Dis..

[45] Rosca C.I., Sharma A., Nisulescu D.-D., Otiman G., Duda-Seiman D.-M., Morariu S.I., Lighezan D.F., Kundnani N.R. (2023). Prevalence of Cardio-Embolic Brain Complications in Permanent and Paroxysmal Atrial Fibrillation Patients. Healthcare.

[46] MacDonell N., Hancox R.J. (2023). Childhood and Adolescent Television Viewing and Metabolic Syndrome in Mid-Adulthood. Pediatrics.

[47] Sharma A., Christodorescu R., Agbariah A., Duda-Seiman D., Dahdal D., Man D., Kundnani N.R., Cretu O.M., Dragan S. (2022). Cardiovascular Risk Prediction Parameters for Better Management in Rheumatic Diseases. Healthcare.

[48] Rosca C.I., Lighezan D.F., Nisulescu D.-D., Sharma A., Neagu M.N., Nistor D., Georgescu D., Kundnani N.R. (2023). Metabolic Syndrome: A Strange Companion of Atrial Fibrillation; A Blessing in Disguise from the Neuropsychiatric Point of View. Biomedicines.

[49] Villarreal S.E.G., Pacheco S.M.S., Alaniz F.V., Conde M.I.B., Pacheco J.M.S., Vazquez K.C.C., Leal A.C.S., Maldonado O.A.T., Contreras J.A.R., Cosain E.I.H. (2023). Risk Factors Associated with COVID-19 Mortality in the State of Durango, Mexico. Int. J. Med. Sci..

[50] Rosca C.I., Kundnani N.R., Tudor A., Rosca M.-S., Nicoras V.-A., Otiman G., Ciurariu E., Ionescu A., Stelian M., Sharma A. (2021). Benefits of prescribing low-dose digoxin in atrial fibrillation. Int. J. Immunopathol. Pharmacol..

[51] Kundnani N.R., Rosca C.I., Sharma A., Tudor A., Rosca M.S., Nisulescu D.D., Branea H.S., Mocanu V., Crisan D.C., Buzas D.R. (2021). Selecting the right anticoagulant for stroke prevention in atrial fibrillation. Eur. Rev. Med. Pharmacol. Sci..

[52] Liu E.N., Yang J.H., Patel L., Arora J., Gooding A., Ellis R., Graves J.S. (2023). Longitudinal analysis and treatment of neuropsychiatric symptoms in post-acute sequelae of COVID-19. J. Neurol..

[53] Bauer K.A., Puzniak L.A., Yu K.C., Klinker K.P., Watts J.A., Moise P.A., Finelli L., Gupta V. (2023). Association of SARS-CoV-2 status and antibiotic-resistant bacteria with inadequate empiric therapy in hospitalized patients: A US multicenter cohort evaluation (July 2019–October 2021). BMC Infect Dis..

[54] Turdybekova Y.G., Kopobayeva I.L., Kamyshanskiy Y.K., Turmukhambetova A.A. (2023). Comparative clinical and placental pathologic characteristics in pregnancies with and without SARS-CoV-2 infection. J. Perinat. Med..

[55] Garabedian C., Vaast P., Bigot J., Sfeir R., Michaud L., Gottrand F., Verpillat P., Coulon C., Subtil D., Houfflin Debarge V. (2014). Esophageal atresia: Prevalence, prenatal diagnosis and prognosis. J. Gynecol. Obstet. Biol. Reprod..

[56] Reppucci M., Kaizer A., Prendergast C., Acker S., Mandell E., Euser A., Diaz-Miron J. (2023). Incidence of congenital complications related to COVID-19 infection during pregnancy. J. Neonatal-Perinatal Med..

[57] Tárnok A., Méhes K. (2002). Gastrointestinal Malformations, Associated Congenital Abnormalities, and Intrauterine Growth. J. Pediatr. Gastroenterol. Nutr..

[58] Doandes F.M., Manea A.-M., Lungu N., Cioboata D., Brandibur T., Costescu O., Hudisteanu A., Boia E.R., Boia M. (2021). Clinical, biological and electroencephalographic monitoring of newborns with neurological risk in the Neonatal Intensive Care Unit. Exp. Ther. Med..

[59] Luxi N., Giovanazzi A., Capuano A., Crisafulli S., Cutroneo P.M., Fantini M.P., Ferrajolo C., Moretti U., Poluzzi E., Raschi E. (2021). COVID-19 Vaccination in Pregnancy, Paediatrics, Immunocompromised Patients, and Persons with History of Allergy or Prior SARS-CoV-2 Infection: Overview of Current Recommendations and Pre- and Post-Marketing Evidence for Vaccine Efficacy and Safety. Drug Saf..

[60] Goldshtein I., Nevo D., Steinberg D.M., Rotem R.S., Gorfine M., Chodick G., Segal Y. (2021). Association Between BNT162b2 Vaccination and Incidence of SARS-CoV-2 Infection in Pregnant Women. JAMA.

[61] Ekingen G., Ceran C., Guvenc B.H., Tuzlaci A., Kahraman H. (2005). Early enteral feeding in newborn surgical patients. Nutrition.

[62] Jorgensen S.C., Tabbara N., Burry L. (2022). A review of COVID-19 therapeutics in pregnancy and lactation. Obstet. Med..

[63] Domnicu A., Mogoi M., Manea A., Boia E.R., Boia M. (2022). Clinical Factors Associated with COVID-19 Severity in Chronic Hospitalized Infants and Toddlers: Data from a Center in the West Part of Romania. Healthcare.

